# Obturator hernia as a cause of small bowel obstruction in an 80-year-old female patient with comorbidities: management and challenges in a resource-limited setting: a case report

**DOI:** 10.1186/s13256-026-06482-1

**Published:** 2026-08-18

**Authors:** Wondwosen Mengist Dereje, Daneal Weldegiorgies Wattaro, Yimenu Wonedimeneh, Kedir Saededin Mehammednure, Hailemariam Yohannes Asefa, Berhanu Shetie Sefene, Mengist Asmamaw Tegegne

**Affiliations:** 1 College of Medicine and Health Sciences, Department of Neurology, University of Gondar, Gondar, Ethiopia; 2 College of Medicine and Health Sciences, Department of Surgery, University of Gondar, Gondar, Ethiopia

**Keywords:** Obturator hernia, Small bowel obstruction, Richter’s herniation, Herniorrhaphy, Case report

## Abstract

**Background:**

Obturator hernia is one of the rarest types of hernia and accounts for only a small percentage of small bowel obstructions. It may be asymptomatic, but most patients present with acute intestinal obstruction. Some patients may also report pain along the medial aspect of the thigh due to compression of the obturator nerve. The clinical presentation of obturator hernia is often non-specific, which makes diagnosis challenging. Imaging modalities such as ultrasonography, herniography, and computed tomography (CT) of the abdomen can aid in diagnosis. Although non-surgical management has been described in the literature, it is frequently associated with high morbidity and mortality. Surgical intervention remains the treatment of choice.

**Case presentation:**

An 80-year-old female patient presented with crampy periumbilical abdominal pain of one week’s duration. She also reported one to two episodes of vomiting per day for five days, consisting of ingested material, associated with failure to pass feces and flatus. She had progressive abdominal distension for four days. Further history revealed a two-week history of high-grade intermittent fever and productive cough with whitish sputum.

On examination, she was in severe abdominal pain and tachycardic, with a pulse rate of 124 beats per minute and an irregularly irregular rhythm. Abdominal examination revealed visible peristalsis. Investigations, including serology and radiologic imaging, were performed. Plain abdominal radiography showed signs of small bowel obstruction, while chest X-ray revealed pneumonia. Electrocardiography demonstrated atrial fibrillation, and echocardiography showed valvular degeneration.

The findings were discussed with the patient and her family, and they opted for surgical intervention. After obtaining informed consent, laparotomy was performed. Intraoperatively, a small bowel loop approximately 50 cm from the ileocecal valve was found herniated through the obturator foramen. A 1 × 2 cm defect was noted on the antimesenteric side of the herniated bowel segment. Repair and anastomosis were performed, and the patient left the operating room with stable vital signs. She remained hospitalized for six days postoperatively and was subsequently discharged with outpatient follow-up. Follow-up visits were uneventful, with significant clinical improvement.

**Conclusion:**

Although obturator hernia is a rare cause of small bowel obstruction, it should be considered in the differential diagnosis of elderly female patients. Maintaining a high index of suspicion facilitates early diagnosis and timely management, as delayed recognition is associated with increased morbidity and mortality.

## Background

Obturator hernias account for 0.07–1% of all hernias and 0.2–1.6% of all cases of mechanical small bowel obstruction [1]. Arnaud de Ronsil first described the obturator hernia in 1724, and Obre performed the first successful surgical repair in 1851 [2].

These hernias most commonly present as acute intestinal obstruction, with the small bowel being the primary content in most cases. Less frequently, the hernia sac may contain the appendix, Meckel’s diverticulum, or omentum. The sac passes through the obturator foramen, following the course of the obturator nerve and muscles. Patients may experience pain along the medial aspect of the thigh [3].

Diagnosis is often delayed and frequently occurs only during laparotomy for bowel obstruction [4]. Strangulation is common, and mortality rates remain high, ranging from 25% to 47.6% [5, 6].

## Case presentation

An 80-year-old female patient was referred from a primary hospital to our tertiary care center due to the unavailability of advanced diagnostic facilities and the absence of a surgical unit. She initially presented to the primary hospital with a one-week history of crampy periumbilical abdominal pain. Two days after the onset of pain, she developed one to two episodes of vomiting of ingested contents, accompanied by failure to pass feces and flatus. She also reported progressive abdominal distension for four days.

The patient was unable to seek earlier medical care because of ongoing armed conflict in her area, which resulted in delayed presentation. Further evaluation revealed a two-week history of high-grade intermittent fever and productive cough with whitish sputum.

She denied any previous similar episodes or known chronic medical illnesses. Following initial assessment, she was promptly referred to our tertiary hospital for further management.

Upon presentation to our hospital, the patient was in severe pain. Her vital signs were as follows: blood pressure 110/70 mmHg, pulse rate 124 beats per minute with an irregularly irregular rhythm, respiratory rate 18 breaths per minute, temperature 37 °C, and oxygen saturation 97% on room air.

**Fig. 1:**
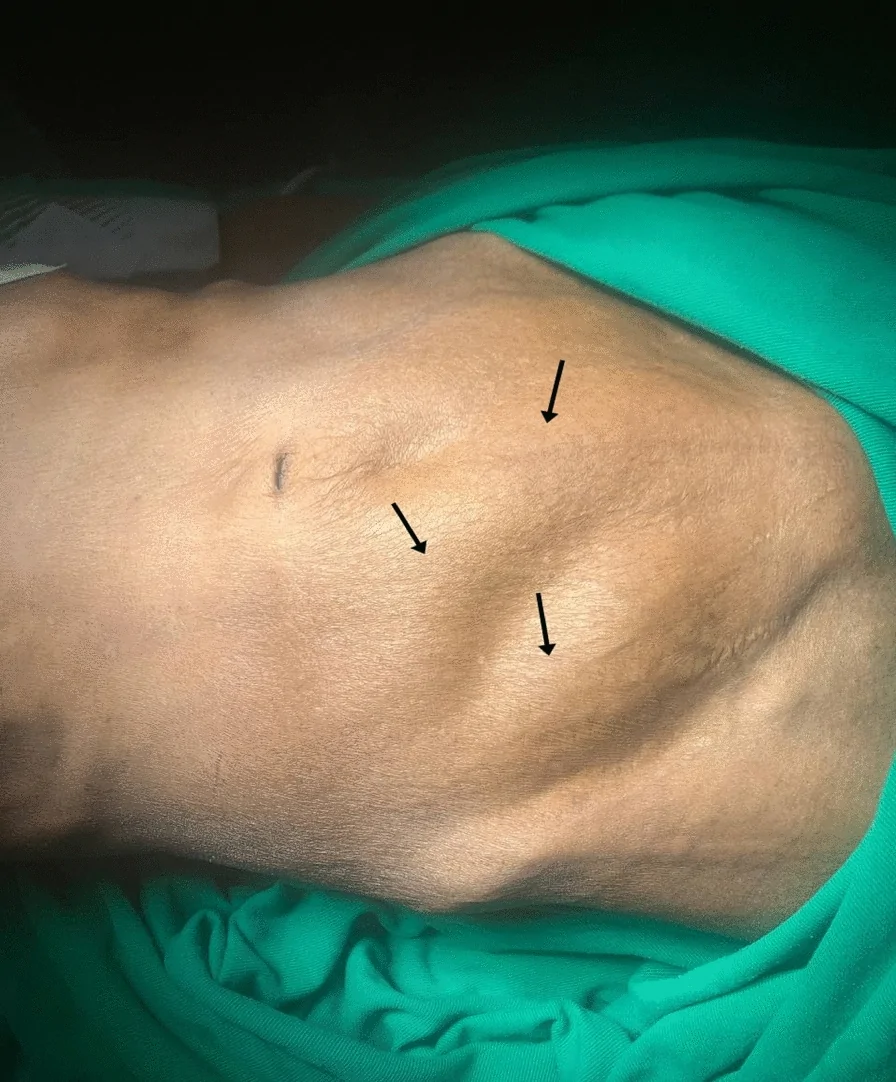
Mild abdominal distension with visible peristalsis (arrow)

Abdominal examination revealed a distended abdomen with visible peristalsis (Fig. 1). On digital rectal examination, anal tone was normal, with minimal stool on the examining finger and no blood or palpable mass detected.

An intravenous line was secured, resuscitation with normal saline was initiated, nasogastric tube decompression was performed, and the patient was sent for imaging studies.

**Fig. 2:**
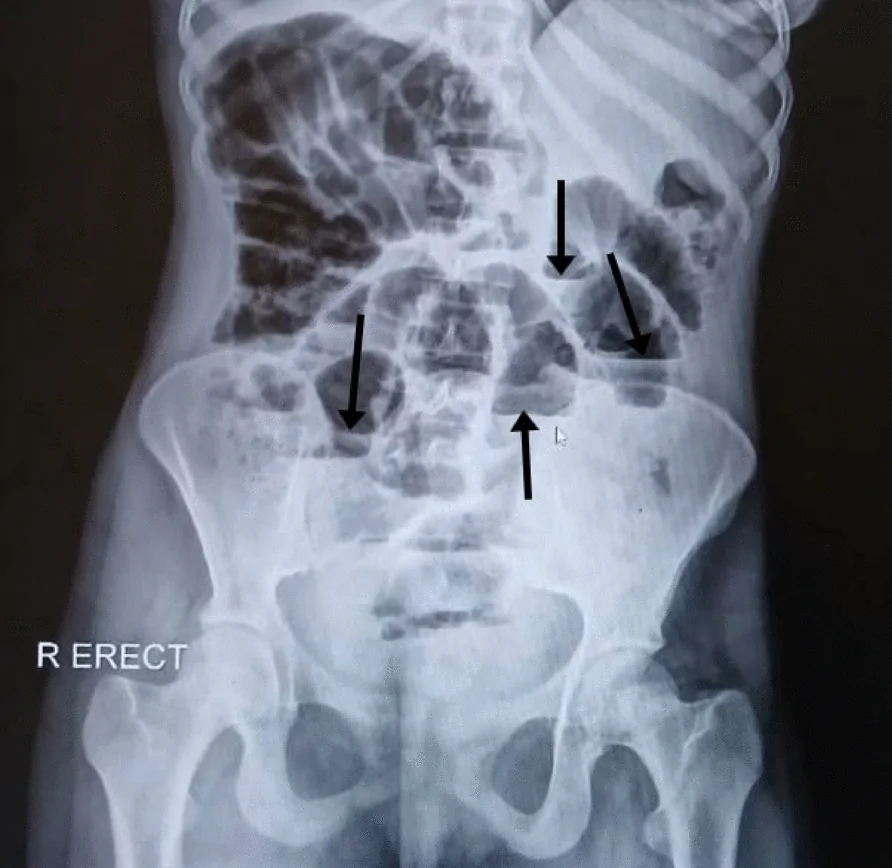
Plain abdominal film sowing multiple central air fluid levels (arrows)

**Fig. 3:**
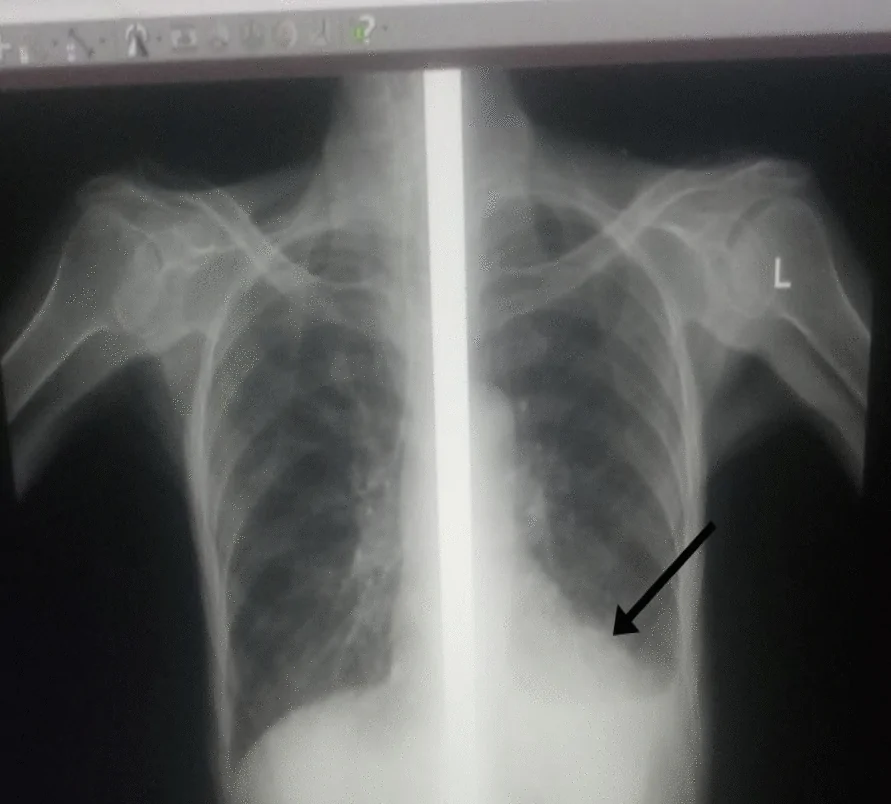
Chest radiography showing left lower lobe consolidation (arrow)

An erect abdominal radiograph demonstrated multiple central air–fluid levels (Fig. 2), suggestive of small bowel obstruction. Chest radiography revealed left lower lobe air-space opacity (Fig. 3), consistent with pneumonia.

CT scan was planned, but there was no functional machine at the time.

The patient was started on intravenous ceftriaxone 1 g, kept nil per os, and placed on maintenance intravenous fluids. While intravenous access was secured, baseline laboratory investigations were obtained. Complete blood count showed leukocytosis (WBC 20 × 10^3^/µL) with neutrophilia (80%) and lymphocytes at 11.4%, hemoglobin 11 g/dL, hematocrit 34%, and platelet count 361 × 10^3^/µL.

Renal function tests revealed serum creatinine 0.27 mg/dL and blood urea nitrogen 27 mg/dL. Liver function tests were within normal limits (alanine aminotransferase 10 U/L, aspartate aminotransferase 24 U/L). Serum albumin was 2.8 g/dL. Electrolyte levels were as follows: sodium 142 mmol/L, potassium 3.85 mmol/L, calcium 2.4 mmol/L, and chloride 108 mmol/L.

**Fig. 4:**
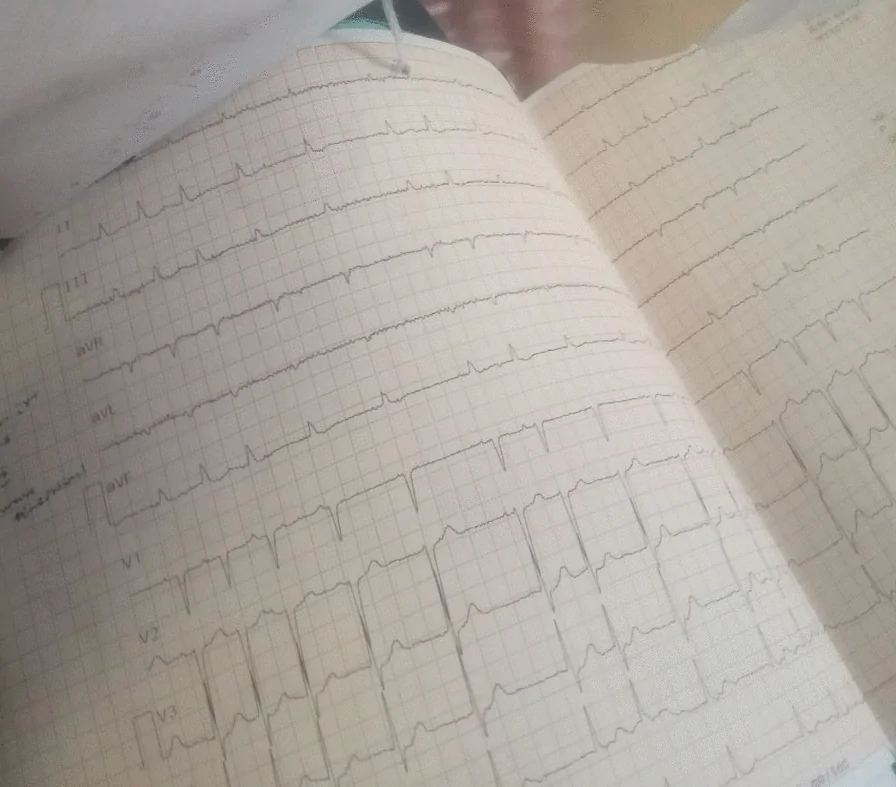
Electrocardiography showing atrial fibrillation

Electrocardiography demonstrated an irregularly irregular rhythm with indistinct P waves (Fig. 4), consistent with atrial fibrillation.

Echocardiography was performed and demonstrated atrial fibrillation with degenerative valvular heart disease, including mild aortic regurgitation and mild mitral regurgitation.

Based on the clinical, laboratory, and imaging findings, the initial diagnosis was small bowel obstruction, likely secondary to primary volvulus, complicated by pneumonia, atrial fibrillation, degenerative valvular heart disease (mitral and aortic regurgitation), and hypoalbuminemia (serum albumin 2.8 g/dL).

The findings and management options were discussed in detail with the patient and her family. Given the patient’s advanced age, multiple comorbidities, and the inherent risks associated with surgical intervention, these concerns were thoroughly explained. Although a laparoscopic approach would have been preferable, it was not available at our institution, and the family was unable to afford referral to another center for the procedure.

After a detailed discussion of the risks and potential complications, both the patient and her family opted for surgical intervention. Intravenous digoxin 0.25 mg was initiated, resulting in a reduction of the heart rate to 104 beats per minute.

**Fig. 5:**
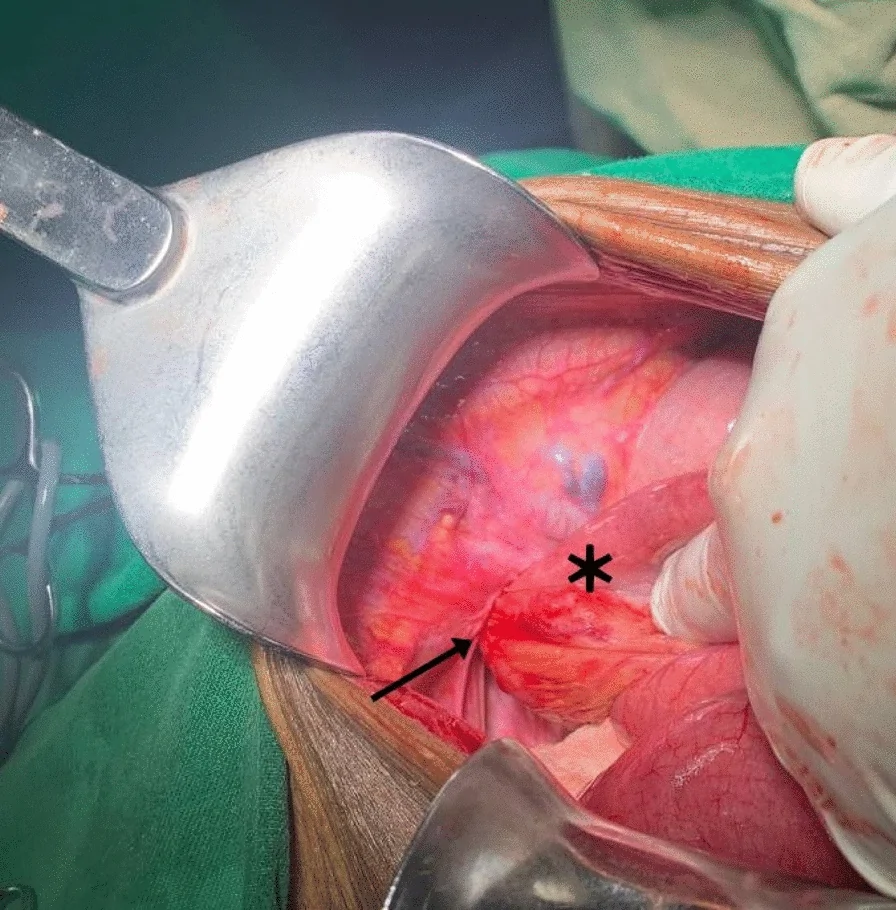
Intraoperative image showing small bowel loop (star) herniated through obturator foramen (arrow)

Following the acquisition of written informed consent, the patient was taken to the operating room for an exploratory laparotomy. After the induction of general anesthesia, the patient was placed in the supine position, and the abdomen was prepared and draped using chlorhexidine and povidone-iodine solution. A midline laparotomy was performed, and the peritoneal cavity was carefully entered. Approximately 200 mL of reactive peritoneal fluid was found over the peritoneum. The reactive peritoneal fluid was completely suctioned. Systematic exploration of the abdominal cavity revealed a segment of small bowel, approximately 50 cm proximal to the ileocecal valve, herniating through the obturator foramen (Fig. 5). The proximal small bowel was edematous, dilated, and twisted 360° in a counterclockwise direction, confirming small bowel volvulus secondary to an obturator hernia. A 1 × 2 cm perforation was identified on the antimesenteric border of the involved bowel segment. The herniated bowel was reduced, followed by repair of the perforation and restoration of bowel continuity with primary intestinal anastomosis. Hemostasis was secured, and the surgical instrument and sponge counts were performed and confirmed to be correct. The procedure was completed without intraoperative complications. The estimated intraoperative blood loss was approximately 100 mL.

After approximately four hours, the abdominal incision was closed in layers, and the patient was transferred to the post-anesthesia care unit in stable condition. She remained in PACU for eight hours. She was subsequently transferred to the recovery unit, stayed there for one day, and was then moved to the surgical ward.

While she was in the PACU and recovery unit, she was maintained on scheduled intravenous analgesics. She began ambulating twelve hours after the operation while still in the recovery unit. After initiating oral feeding, she preferred oral analgesics and was switched to paracetamol, with adequate pain control achieved.

Wound care was initiated 24 h after the procedure and was performed daily throughout her hospital stay. The wound continued to heal well, with no signs of complications, and was in good condition at the time of discharge. After a six-day stay in our hospital, her intravenous antibiotics were transitioned to oral antibiotics. She was linked to a nearby health center for continued wound care, which was provided for an additional eight days. Suture removal was performed twelve days after the operation.

At her two-week follow-up visit in our outpatient clinic, the wound was healing well, and she was comfortable without requiring any analgesics.

During her fourth-week follow-up visit, the wound had completely healed. All vital signs were within the normal range, including her pulse rate of 82 beats per minute. Follow-up investigations were also within normal limits. She showed significant clinical improvement and had resumed her social activities, including visiting her granddaughters and grandsons.

At her eighth-week follow-up visit, the patient had continued to show significant improvement in her functional status. She had resumed engaging in simple daily activities, including cooking and preparing coffee, and expressed motivation to gradually return to her other routine activities.

No postoperative complications were observed, and no adverse drug reactions were reported. No postoperative complications occurred (Clavien–Dindo Grade 0/no reportable complication).

During outpatient follow-up visits conducted at two, four, and eight weeks after discharge, the patient demonstrated continued clinical improvement. She reported no new complaints, and there was no evidence of recurrent abdominal symptoms. She was also referred to the medical team for ongoing evaluation and management of her atrial fibrillation and associated cardiovascular conditions. Overall, the patient had a favorable recovery following surgical management of small bowel volvulus secondary to an obturator hernia.

The overall clinical timeline is summarized in Table 1.

**Table 1:** The overall clinical course of the patient

Time	Clinical events
2 weeks before admission	High-grade intermittent fever and productive cough with whitish sputum developed
1 week before admission	Crampy periumbilical abdominal pain began
5 days before admission	1–2 episodes of vomiting of ingested contents and failure to pass feces and flatus developed
4 days before admission	Progressive abdominal distension developed
Day 0 – Presentation at tertiary hospital	Referred from primary hospital because advanced diagnostics and surgical services were unavailable; delayed presentation due to armed conflict
Initial assessment	Severe pain; BP 110/70 mmHg, pulse 124/min irregularly irregular, RR 18/min, T 37 °C, SpO₂ 97%; distended abdomen with visible peristalsis; DRE normal tone, minimal stool, no blood/mass
Emergency management	IV access, normal saline resuscitation, nasogastric decompression, nil per os, imaging requested
Investigations	Abdominal X-ray: multiple central air–fluid levels suggesting SBO; chest X-ray: left lower lobe pneumonia; CT unavailable. CBC: WBC 20 × 10^3^/µL (80% neutrophils), Hb 11 g/dL, platelets 361 × 10^3^/µL. Albumin 2.8 g/dL; renal, liver function, and electrolytes largely acceptable. ECG: atrial fibrillation. Echocardiography: AF with mild aortic and mitral regurgitation
Diagnosis	Small bowel obstruction, initially suspected primary volvulus, with pneumonia, atrial fibrillation, degenerative valvular heart disease, and hypoalbuminemia
Preoperative optimization	Counseling and informed written consent obtained. IV ceftriaxone and IV digoxin 0.25 mg administered; heart rate decreased to 104/min
Operation (Day 0)	Exploratory laparotomy. Approximately 200 mL reactive peritoneal fluid aspirated. Obturator hernia with incarcerated small bowel 50 cm proximal to ileocecal valve causing 360° counterclockwise volvulus identified. A 1 × 2 cm antimesenteric perforation found. Repair and anastomosis performed. Surgery lasted ~ 4 h without intraoperative complications
Immediate postoperative period	8 h in PACU, followed by 1 day in recovery unit, then transfer to surgical ward
Postoperative day 1	Clear oral fluids initiated 24 h after surgery and advanced as tolerated
Postoperative days 2–6	Received IV antibiotics, wound care, and close monitoring. Stayed 4 days in surgical ward (total postoperative stay: 6 days)
Discharge	Discharged in stable condition with outpatient surgical follow-up and referral for ongoing atrial fibrillation management
2-week follow-up	Significant clinical improvement; no new complaints
4-week follow-up	Continued recovery without new complaints
8-week follow-up	Remained clinically well with no new complaints; continued medical follow-up for atrial fibrillation

## Strength and limitation

Although the patient was successfully managed, this case is not without limitations. The strengths and limitations of this case report are described below.

### Strengths

- Obturator hernia is one of the rarest causes of bowel obstruction. This case provides valuable insight for clinicians worldwide by highlighting the importance of considering obturator hernia as a potential differential diagnosis in patients presenting with bowel obstruction.
- Despite the patient's advanced age, multiple comorbidities, and resource limitations, including the lack of advanced imaging modalities, the patient was successfully managed with an excellent clinical outcome. Referral was considered for imaging but it was not possible due to economic hardship.

### Limitations

- Elderly patients presenting with bowel obstruction often require advanced imaging to exclude other potential causes, such as bowel obstruction secondary to malignancy. In this case, advanced imaging should ideally have been performed. When such imaging is unavailable, timely referral to a better-equipped healthcare facility should be considered.
- Although the patient continues regular cardiac follow-up at the same hospital and has been instructed to return to the surgical clinic if any symptoms or concerns develop, the relatively short duration of surgical follow-up represents another limitation of this case report.

## Discussion

Obturator hernia is an extremely rare type of pelvic and intra-abdominal hernia. The hernia sac protrudes through the obturator foramen, which is located bilaterally in the anterolateral pelvic wall. It is most commonly observed in elderly, frail women. This female predominance has been attributed to pregnancy, which relaxes the pelvic peritoneum and results in a wider, more horizontal obturator canal [7].

The hernia occurs three times more frequently on the right side than on the left and may rarely coexist with bilateral or femoral hernias, as documented in the literature [8]. In the reported case, the hernia was on the right side, consistent with the common site described in previous studies.

Often referred to as the “little old lady’s hernia,” it typically affects multiparous and elderly, emaciated women. The condition is nine times more common in females due to a wider pelvis, a more triangular obturator canal opening, and a greater transverse diameter. Additional risk factors include chronic obstructive pulmonary disease, chronic constipation, and ascites [3].

Obturator hernias are typically asymptomatic until bowel obstruction occurs, presenting as acute or intermittent small bowel obstruction. In a classic case, patients may report pain along the medial aspect of the thigh, which worsens with hip or thigh movement due to compression of the obturator nerve [9].

This manifestation, known as the Howship–Romberg sign, is observed in only 15%–50% of cases [10]. Another sign, the Hannington-Kiff sign—characterized by an absent adductor reflex in the thigh—is more specific but much less commonly seen [10].

Symptoms are often vague, including nausea, vomiting, or other signs of bowel obstruction such as abdominal pain and constipation. Literature indicates that up to 80% of patients with obturator hernias exhibit symptoms of bowel obstruction, which is frequently partial because a high proportion involve Richter’s herniation of the bowel into the obturator canal [1].

This often results in a clinical picture of intermittent bowel obstruction, which is an important feature to elicit from the patient’s history for an accurate diagnosis (1). Various investigative modalities can aid in diagnosis, including ultrasonography, herniography, and computed tomography (CT) of the abdomen. Among these, CT has the highest specificity and sensitivity [10] and is recommended for patients with suspected obturator hernia or unexplained intestinal obstruction. The accuracy of CT in diagnosing obturator hernia is approximately 90% [11, 12], with even higher reliability in cases of strangulated hernias. CT may demonstrate small bowel loops located behind the pectineus muscle or the presence of air in the subpubic canal [13].

CT imaging that shows bowel herniating through the obturator foramen and positioned between the pectineus and obturator muscles is considered the most reliable diagnostic indicator [14]. However, caution is advised against over-reliance on imaging, as definitive diagnosis can often only be confirmed intraoperatively [14].

In the reported case, a CT scan was not performed due to its unavailability, and the diagnosis was made intraoperatively.

Surgery is the only definitive treatment for obturator hernia. Various operative approaches have been described, including inguinal, retropubic, transperitoneal [15, 16], abdominal, and laparoscopic techniques [1].

In emergency settings, the abdominal approach via a low midline incision is most commonly preferred, as it provides adequate exposure of the obturator ring and allows identification and resection of any ischemic bowel. In this case, laparotomy through a low midline incision was performed. More recently, laparoscopic surgery has emerged as an alternative approach for obturator hernia repair [17].

The advantages of laparoscopic surgery include reduced postoperative pain, shorter hospital stay, and lower complication rates. However, it is generally reserved for non-strangulated hernias due to its technical complexity and longer learning curve. Both transabdominal and extraperitoneal laparoscopic approaches have been described. The transabdominal approach is particularly suitable in emergency settings, as it allows full exploration of the abdominal cavity, identification of the cause of bowel obstruction, reduction of the hernia, thorough inspection for ischemic bowel, and resection if necessary [18].

The laparoscopic total extraperitoneal (TEP) approach is particularly feasible when the diagnosis is established preoperatively in symptomatic patients. Frequently, obturator hernias are detected incidentally during TEP repair for inguinal hernias, highlighting the importance of inspecting all myopectineal orifices during this procedure to identify and repair asymptomatic obturator hernias [16].

In the reported case, although laparoscopic surgery would have been preferable due to the absence of strangulation, it was not performed because of unavailable laparoscopic facilities and the absence of preoperative CT imaging. Therefore, a midline incision and laparotomy were performed, and the diagnosis was made intraoperatively.

Repair techniques include simple suture closure, closure of the obturator with adjacent tissue, or mesh placement during laparotomy [19].

Many authors prefer simple closure of the hernial defect using one or more interrupted sutures, particularly when bowel resection is required [15, 17]. In the reported case, we performed a simple suture closure, and the outcome was satisfactory.

The most challenging aspect of managing our patient was the presence of multiple comorbidities, including pneumonia, atrial fibrillation, valvular heart disease, and hypoalbuminemia. In patients with such comorbidities, it is crucial to stabilize and treat underlying conditions prior to surgical intervention, and less invasive procedures, such as laparoscopic surgery, should be considered to optimize outcomes.

Surgical management remains the mainstay of treatment for obturator hernia. Although non-surgical approaches have been described, they are generally associated with high morbidity and mortality [20] and should only be considered when the patient is unfit for surgery. In our case, despite the patient’s advanced age and comorbidities, the preoperative diagnosis was not established, making surgical management the most appropriate option.

## Conclusion

This case highlights obturator hernia as a rare cause of bowel obstruction that is often overlooked because of its uncommon occurrence. In addition to its rarity, the absence of specific clinical signs and symptoms frequently leads to delayed diagnosis, which may result in poorer patient outcomes. Therefore, obturator hernia should be considered in the differential diagnosis of patients presenting with signs and symptoms of bowel obstruction. Early recognition and timely surgical intervention are essential for improving patient outcomes.

### Patient’s perspective

At first, I believed my cough and fever were due to a simple common cold and was not concerned. However, when I developed severe abdominal pain, abdominal distension, and was unable to pass stool or flatus, I realized my condition was serious. Because of the ongoing war, I was unable to seek medical care immediately and was uncertain whether I would safely reach the hospital. Although I was 80 years old, I had remained physically active before my illness. After being referred to a tertiary hospital, I was informed of my diagnosis and the risks associated with surgery, particularly given my multiple comorbidities. Despite these concerns, I agreed to the operation, which was successful. I am grateful to my family and the healthcare professionals for their care and support. My symptoms have resolved, and I have returned to my usual daily activities while continuing regular cardiac follow-up.

## Data Availability

Due to privacy of the patient the authors decline to share data. But the data will be presented upon reasonable request.
